# Supplementary material for: A UMLS-based spell checker for natural language processing in vaccine safety
Source: BMC Med Inform Decis Mak. 2007 Feb 12;7:3. doi: 10.1186/1472-6947-7-3 (PMC1805499; doi:10.1186/1472-6947-7-3)
Supplement: Additional file 13 — RAP application directory. Sets up directory for RAP (RDF application for PHP) source code [file 1472-6947-7-3-S13.gz › rap/doc/phpdoc/dataset/NamedGraphDb.html]

xml version="1.0" encoding="iso-8859-1"?


Docs For Class NamedGraphDb


## Class NamedGraphDb

Description

Description |
Methods (details)

Persistent NamedGraph implementation that extends a DbModel.

The graphName is not stored in the database. As soon as the namedGraph is added to a RDF dataset the graphName is saved.

History:

- access: public
- author: Daniel Westphal (http://www.d-westphal.de)
- version: V0.9

Located in /dataset/NamedGraphDb.php (line 20)

```
Object
   |
   --Model
      |
      --DbModel
         |
         --NamedGraphDb
```

Method Summary

Description |
Methods (details)

NamedGraphDb
NamedGraphDb
(ADODBConnection &$dbConnection, string $modelURI, string $modelID, string $graphName, [mixed $baseURI = NULL])

string
getGraphName
()

void
setGraphName
(string $graphName)

Methods

Description |
Methods (details)

Constructor NamedGraphDb (line 42)

Constructor Do not call this directly.

Use the method getModel,getNewModel or putModel of the Class NamedGraphDb instead.

- access: public

NamedGraphDb

NamedGraphDb
(ADODBConnection &$dbConnection, string $modelURI, string $modelID, string $graphName, [mixed $baseURI = NULL])

- ADODBConnection
  &$dbConnection
- string
  $modelURI
- string
  $modelID
- string
  $graphName

getGraphName (line 68)

Return the graph name.

- access: public

string

getGraphName
()

setGraphName (line 57)

Sets the graph name.

- access: public

void

setGraphName
(string $graphName)

- string
  $graphName

#### Inherited Methods


Inherited From DbModel

> DbModel::DbModel()  
> DbModel::add()  
> DbModel::addModel()  
> DbModel::addNamespace()  
> DbModel::addParsedNamespaces()  
> DbModel::addWithoutDuplicates()  
> DbModel::close()  
> DbModel::contains()  
> DbModel::containsAll()  
> DbModel::containsAny()  
> DbModel::delete()  
> DbModel::equals()  
> DbModel::find()  
> DbModel::findCount()  
> DbModel::findFirstMatchingStatement()  
> DbModel::findRegex()  
> DbModel::findVocabulary()  
> DbModel::getMemModel()  
> DbModel::getParsedNamespaces()  
> DbModel::intersect()  
> DbModel::isEmpty()  
> DbModel::rdqlQuery()  
> DbModel::rdqlQueryAsIterator()  
> DbModel::reify()  
> DbModel::remove()  
> DbModel::removeNamespace()  
> DbModel::replace()  
> DbModel::saveAs()  
> DbModel::setBaseURI()  
> DbModel::size()  
> DbModel::subtract()  
> DbModel::toString()  
> DbModel::toStringIncludingTriples()  
> DbModel::unite()  
> DbModel::writeAsHtml()  
> DbModel::writeAsHtmlTable()  
> DbModel::writeRdfToString()

Inherited From Model

> Model::Model()  
> Model::findForward()  
> Model::getBaseURI()  
> Model::getMemModelByRDQL()  
> Model::getOntModel()  
> Model::getResmodel()  
> Model::load()  
> Model::visualize()

Inherited From Object

> Object::toString()

Documentation generated on Thu, 7 Jul 2005 13:42:15 +0200 by phpDocumentor 1.3.0RC3
